# Alterations in the self-renewal and differentiation ability of bone marrow mesenchymal stem cells in a mouse model of rheumatoid arthritis

**DOI:** 10.1186/ar3098

**Published:** 2010-07-22

**Authors:** Sindhu T Mohanty, Lucksy Kottam, Alessandra Gambardella, Martin J Nicklin, Les Coulton, David Hughes, Anthony G Wilson, Peter I Croucher, Ilaria Bellantuono

**Affiliations:** 1 Mellanby Centre for Bone Research, Department of Human Metabolism, Beech Hill Rd, University of Sheffield, Sheffield S10 2RX, UK; 2 Department of Infection and Immunity, Beech Hill Rd, University of Sheffield, Sheffield, S10 2RX, UK

## Abstract

**Introduction:**

Rheumatoid arthritis (RA) is a chronic systemic autoimmune disease primarily involving the synovium. Evidence in recent years has suggested that the bone marrow (BM) may be involved, and may even be the initiating site of the disease. Abnormalities in haemopoietic stem cells' (HSC) survival, proliferation and aging have been described in patients affected by RA and ascribed to abnormal support by the BM microenvironment. Mesenchymal stem cells (MSC) and their progeny constitute important components of the BM niche. In this study we test the hypothesis that the onset of inflammatory arthritis is associated with altered self-renewal and differentiation of bone marrow MSC, which alters the composition of the BM microenvironment.

**Methods:**

We have used Balb/C Interleukin-1 receptor antagonist knock-out mice, which spontaneously develop RA-like disease in 100% of mice by 20 weeks of age to determine the number of mesenchymal progenitors and their differentiated progeny before, at the start and with progression of the disease.

**Results:**

We showed a decrease in the number of mesenchymal progenitors with adipogenic potential and decreased bone marrow adipogenesis before disease onset. This is associated with a decrease in osteoclastogenesis. Moreover, at the onset of disease a significant increase in all mesenchymal progenitors is observed together with a block in their differentiation to osteoblasts. This is associated with accelerated bone loss.

**Conclusions:**

Significant changes occur in the BM niche with the establishment and progression of RA-like disease. Those changes may be responsible for aspects of the disease, including the advance of osteoporosis. An understanding of the molecular mechanisms leading to those changes may lead to new strategies for therapeutic intervention.

## Introduction

Rheumatoid arthritis (RA) is a systemic autoimmune disease characterised by chronic disruptive polyarthritis. There is no cure for RA and today's treatments aim at achieving the lowest possible level of arthritis disease activity. In the UK alone RA costs the health system around £560 million a year, and £1.8 billion a year in absence from work. The events preceding the disease and leading to its initiation and progression are unknown. Thus, an understanding of these processes is crucial for the identification of new and more cost-effective treatments and for the move from a manageable to a curable disease.

Although the main disease site is the synovium, there is growing evidence that the bone marrow (BM) is actively involved and may even be the primary initiating site of the disease [1]. Abnormalities in both the haemopoietic progenitor cells and the BM stroma have been described [2]. Patients with active RA have been seen to exhibit low frequency and accelerated apoptosis of BM CD34+ cells and defective clonogenic potential [2]. Difficulties in haemopoietic stem cell (HSC) transplantation with very common, but unfortunately, relatively short-lived responses have been reported [3]. Moreover, age-inappropriate shortening of telomeres in CD4 T cells and granulocytes have been shown not only in patients affected by RA but also in healthy adult HLA-DR4 donors, the HLA haplotype with the major genetic risk factor for RA [4]. These data suggest that HSC undergo increased replicative stress not only in patients affected by RA but also in patients predisposed to RA and it can occur independently of the rheumatoid disease process. The causes of the increased apoptosis and replicative stress of HSC in patients affected by RA are unknown.

HSC requires a balanced supportive environment, the niche, to survive, proliferate and appropriately differentiate. An impaired hematopoiesis-supporting capacity of BM stroma has been described in RA patients [2]. However, it is unknown what the changes are in the bone marrow (BM) niche, which may lead to ineffective support of HSC. Mesenchymal stem cells (MSCs), and their differentiated progenies, including osteoblasts and adipocytes, have been shown to be important elements of the BM niche and have a role in HSC survival, proliferation and differentiation [5-8]. Cells with properties of MSC have been found to increase in the synovium of patients affected by RA and are thought to be one of the main causes of perpetuation of the disease, suggesting a possible dysregulation of MSC proliferative capacity [9]. In this study, we hypothesize that bone marrow MSCs undergo enhanced self-renewal at the onset of RA-like disease.

To test this hypothesis we have used an Interleukin-1 receptor antagonist knock out mouse model of RA on a Balb/c background (Balb/c IL1ra-/-), which spontaneously develops inflammatory arthropathy with inflammatory cellular infiltration, pannus formation and periarticular bone erosions, similar to RA in humans, with 100% penetrance by weeks 20 of age [10]. We have determined the changes present in the bone marrow microenvironment before disease onset and with development of RA-like disease.

This study showed the presence of important changes occurring in the bone marrow niche before disease onset in relation to mesenchymal progenitors, adipogenesis and osteoclastogenesis. It identifies progressive amplification of mesenchymal progenitors in the bone marrow with disease onset and progression but impairment of their differentiation to the osteogenic lineage.

## Materials and methods

### Reagents and animals

Balb/c and C57BL6 Interleukin-1 receptor antagonist knock-out mice (Balb/c IL-1ra-/- and C57Bl/6 IL1ra-/- respectively) and age-matched wild type (WT) controls were used for the experiments. All animal procedures were conducted in compliance with institutional and national guidelines and with ethics committee approval (PPL 40/2655). Balb/c IL-1ra-/- mice spontaneously develop chronic inflammatory arthropathy that closely resembles RA in humans [10]. IL-1ra-/- mice were monitored by visual inspection and mice were sacrificed a) before disease onset; b) at disease onset identified by the first visual appearance of swelling and redness of joints; or c) late disease, characterized by persistent disease (five weeks from the first sight of joint swelling). In contrast C57BL6 IL1 ra-/- do not develop any inflammatory arthropathy [11] and were used as a further control group. Bone marrow cells (BMCs) were flushed from the long bones of mice and used for progenitor assays. Tibiae were dissected from soft tissue and used for microCT and histomorphometric analysis.

### Progenitor cell assays

#### Colony Forming Unit-Fibroblasts (CFU-F)

BMCs were plated at the densities of 5 × 10^5 ^and 10^6 ^cells per well in duplicate in murine-mesenchymal stem cell complete medium (Stem Cell Technologies, Vancouver, Canada). The plates were incubated for 14 days at 37°C in 5% CO_2 _in air. Colonies were visualized by Giemsa's stain solution (VWR, Leicestershire, UK). Colonies with a minimum of 50 cells were considered as one CFU-F.

#### Colony Forming Unit-Osteoblast (CFU-O)

BMCs were plated at a density of 5 × 10^5 ^and 10^6 ^cells per well in duplicate in Dulbecco's modified Eagle medium (DMEM) plus 10% fetal calf serum (FCS) supplemented with 100 nM dexamethasone (Sigma Aldrich, St. Louis, MO, USA), 10 mM β glycerophosphate (Sigma Aldrich) and 0.05 mM L-Ascorbic Acid (Sigma Aldrich). Cells were maintained in culture for 14 days and fed twice a week. Cells were stained for alkaline phosphatase (ALP) activity using the 86 R alkaline phosphatase kit (Sigma Aldrich). The colonies comprising at least 20 cells positive for ALP were considered as one CFU-O.

#### Colony Forming Unit-Adipocyte (CFU-A) by limiting dilution analysis

BMCs were plated at limiting dilutions (range 10^5 ^to 6.25 × 10^3 ^cells, eight wells/dilution) in DMEM plus 10% FCS (Hyclone, Fisher Scientific, Loughborough, UK) in a 96-well culture plate and fed twice a week for two weeks. After two weeks cultures were supplemented with Rosiglitazone (5 μM) and cultured at 37°C in CO_2 _in air for two more weeks. To detect lipid vacuoles cultures were stained for Oil red O as previously described [12]. This method follows the Poisson distribution and measures the abundance of cells able to perform a particular function, in this case to become adipocytes. A well is considered positive if it contains more than 20 cells with red lipid vacuoles. The number of CFU-A was calculated using the formula Fo = e^-x^, where Fo is the fraction of colony-negative wells, *e *is the constant whose value is 2.71 and *x *is the number of colony forming units per well [13].

### Micro computed tomography (CT)

The tibiae were scanned using a microCT scanner (model 1172, Skyscan, Aartselaar, Belgium) at 50 kV and 200 μA with a 0.5 aluminium filter using a detection pixel size of 4.3 μm. Images were captured every 0.7° through 180° rotation of the bone. The scanned images were reconstructed using the Skyscan Recon software and analysed using Skyscan CT analysis software. A standard trabecular bone volume of interest was chosen starting 0.2 mm from the growth plate and included all the trabeculae in 1 mm^3 ^of bone.

### Histological analysis

Tibiae were fixed in 10% neutral buffered formalin, decalcified in EDTA, and embedded in paraffin, and 3 μm sections were cut using a Leica Microsystems microtome (Leica Microsystems, Milton Keynes, UK). The sections were stained with either Hematoxylin and Eosin or Tartrate Resistant Acid Phosphatise (TRAP) to identify osteoclasts and counterstained with Gill's hematoxylin. The sections were examined by light microscopy (Leica Microsystems). The number of osteoblasts and osteoclasts per millimeter were measured on 6.5 mm of the corticoendosteal surfaces, starting 0.25 mm from the growth plate using the Osteomeasure analysis software (Osteometrics, Decatur, GA, USA). Bone marrow adiposity was determined by measuring the area occupied by the adipocytes recognised as white holes defined by the light blue staining cell wall over 0.75 mm^2^.

### Statistical analysis

All experiments were analyzed using a t-test or a one-way ANOVA-Dunnett's post test for multiple comparisons. All results are expressed as the mean ± SEM. Significant *P-*values were less than 0.05.

## Results

### Decreased number of bone marrow progenitors with adipogenic potential and bone marrow adiposity precedes the onset of RA disease

**Figure 1:**
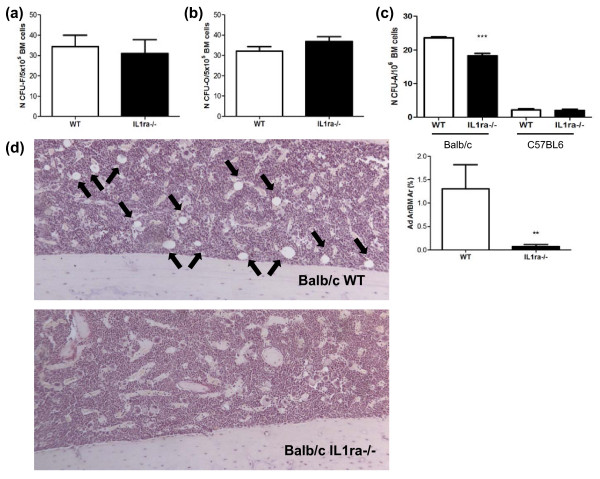
**Decreased bone marrow progenitors with adipogenic potential and adiposity before RA-like disease onset**. No significant difference was found in the number of CFU-F **(a) **(*n *= 8) and CFU-O **(b) **(*n *= 12) in Balb/c IL-1ra-/- mice when compared to age-matched (age 5 to 12 weeks) wild type mice before disease onset. A significant decrease in CFU-A was found in Balb/c IL-1ra-/- mice (*n *= 8) but not in C57BL/6 IL-1ra-/- mice **(c) **(*n *= 6) when compared to their respective age matched wild type mice. **(d) **A representative histological section of tibia showing absence of adipocytes in the bone marrow of Balb/c IL1ra-/- and representation of the average percentage of bone marrow area covered by adipocytes in Balb/c IL1ra-/- mice and age matched WT controls (*n *= 9). Data were analysed by Student t-test. *** *P *< 0.001. WT, wild type mice; CFU-F, colony forming unit fibroblast; CFU-O, Colony forming unit osteoblast; CFU-A, colony forming unit adipocyte; BM, bone marrow; Ad Ar, adipocyte area; BM Ar, bone marrow area; Oc, osteoclasts; Ob, osteoblasts; BV, bone volume; TV, tissue volume. Black arrows indicate presence of adipocytes.

To determine whether there were changes in bone marrow MSC, which preceded the onset of RA-like disease we determined the number of mesenchymal progenitors in Balb/c IL1 ra-/- and age matched wild type mice (age 5 to 12 weeks). Balb/c IL1ra-/- mice were sacrificed when their joints did not show any visual sign of swelling and bone marrow cells were harvested to measure the number of mesenchymal progenitors. Subsequent histological analysis of the joints showed a normal synovial membrane with only occasional modest cellular inflammatory infiltration but no pannus formation or cartilage and bone erosion (data not shown). No difference in the number of mesenchymal progenitors (CFU-F, Figure 1a, *n *= 8, *P *= 0.72) and progenitors with osteogenic potential (CFU-O, Figure 1b, *n *= 12, *P *= 0.17) was found in these mice. However, a significant decrease in the number of progenitor with adipogenic potential (Figure 1c, CFU-A, *n *= 8, *P *< 0.0001) was detected. This was not uniquely due to the deletion of the IL1 receptor antagonist gene in this mouse model because no significant difference in the number of CFU-A was found in the bone marrow of C57BL6 IL1ra-/- mice, which have the same genetic deletion but do not develop RA-like arthritis (Figure 1c, *n *= 6, *P *= 0.85). These data suggest that mice predisposed to develop RA-like arthritis have a deficiency in mesenchymal progenitors with adipogenic potential.

To determine if the decrease in mesenchymal progenitors with adipogenic potential resulted in a decrease in bone marrow adiposity, the percentage of bone marrow area occupied by adipocytes was determined in Balb/c IL1ra-/- and compared to age matched wild type controls (age 6 to 14 weeks). As expected this was significantly reduced (Figure 1d, *n *= 9, *P *= 0.02).

### Decreased numbers of activated osteoclasts precede the onset of RA disease

**Figure 2:**
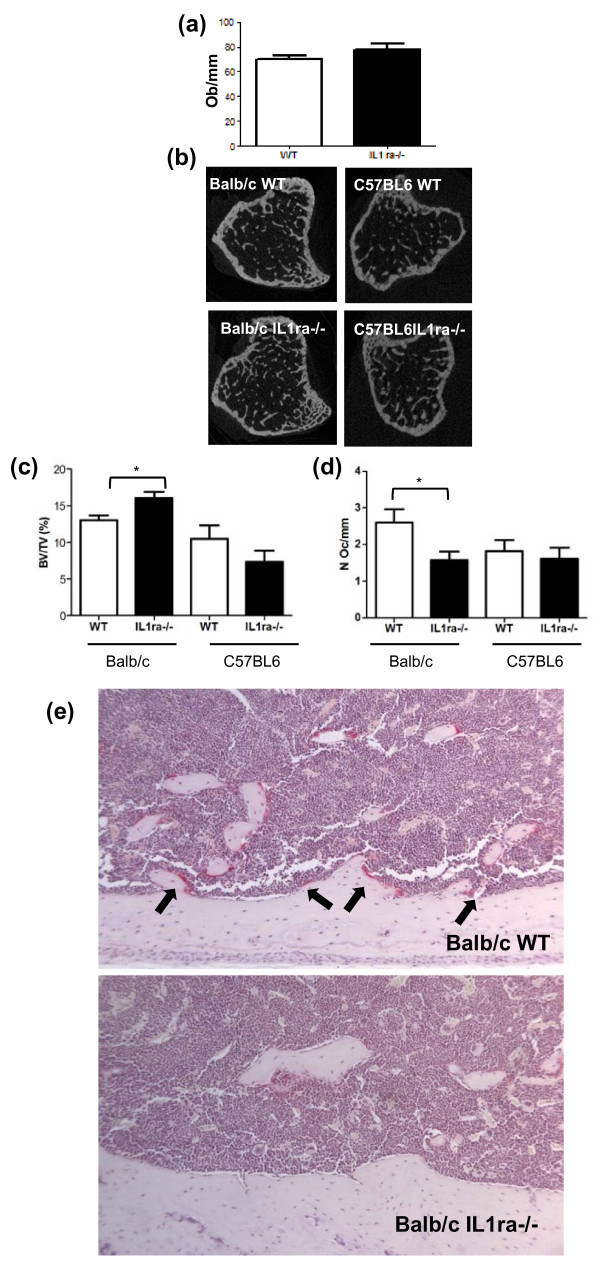
**Decreased osteoclasts activity precedes the onset of RA disease**. **(a) **No difference in the number of osteoblasts/mm over 6.5 mm of tibial cortico-endosteal surface in Balb/c IL1ra-/- compared to WT age matched controls before disease onset (*n *= 8). **(b) **A representative example of a horizontal section of tibia after X-ray micro-CT scanning of a Balb/c IL1ra-/- and a C57BL6 IL1ra-/- mouse and respective age matched WT control. **(c) **X-ray micro CT scanning of tibia of Balb/c IL1ra-/- mice showed significant increase in bone volume in the cancellous bone area compared to age matched WT control (*n *= 9). In contrast, no significant difference was found in C57BL6 IL1ra-/- mouse when compared to their age matched WT control (*n *= 6). **(d) **No significant difference in osteoclast numbers over 6.5 mm of tibial cortico-endosteal surface was observed in C57BL/6 IL-1ra-/- mice and their age-matched WT controls (*n *= 6). **(e) **A representative histological section of tibia showing decreased number of osteoclasts at the cortico-endosteal surface in Balb/c IL1ra-/- mice compared to WT age matched control and a graphic representation of the data showing a significant decrease in osteoclast numbers over 6.5 mm of tibial cortico-endosteal surface in Balb/c IL-1ra-/- mice compared to age matched wild type control mice (*n *= 8). All data are presented as means ± SEM and analysed by unpaired t-test. *P < 0.05. WT, wild type mice. Black arrows indicate presence of TRAP+ osteoclasts.

As adipogenesis and osteogenesis are often inversely correlated, quantification of bone mass and the number of osteoblasts was carried out. The number of osteoblasts was determined by histomorphometric analysis of the tibia of Balb/c IL1 ra-/- mice (five to seven weeks of age) and compared to WT age-matched controls. No difference in the number of osteoblasts at the endocortical surface was found (Figure 2a, *n *= 8, *P *= 0.17). In the contrast analysis of a 1 mm^3 ^region of trabecular bone in the tibia of Balb/c IL1ra-/- mice, carried out by microCT analysis, an average 23% increase in bone mass in Balb/c IL1ra-/- mice was revealed when compared to age-matched WT controls (age 5 to 12 weeks, Figure 2b,c, *n *= 9, *P *= 0.01). This was the result of an increase in trabecular number (3.23 ± 0.14 vs 3.81 ± 0.19 WT vs Balb/C IL1ra-/-, *n *= 9, *P *= 0.03) and not trabecular thickness (data not shown). To assess that this change in bone mass was not simply due to the deletion of an IL1 receptor antagonist gene, C57BL6 IL1ra-/- mice (aged 11 to 12 weeks), which harbour the same deletion but do not develop RA-like arthritis, were also analysed. No significant difference in bone mass was found when compared to the age-matched WT controls and, if anything, a trend to decrease was observed (Figure 2b, c, *n *= 6, *P *= 0.21). As osteoclasts are responsible for the resorption of bone, the increase in bone mass is most likely due to a decrease in the number of activated osteoclasts. Therefore, the number of TRAP+ osteoclasts at the endocortical surface was scored and seen to significantly decrease (Figure 2d, e, *n *= 8, *P *= 0.03). This was not seen in C57BL6 IL1ra-/- mice (Figure 2d, *n *= 6, *P *= 0.62). These data suggest that the increase in bone mass seen in Balb/c IL1 ra-/- is due to decreased maturation and activation of osteoclasts.

### Bone marrow mesenchymal progenitors increase in numbers with onset and progression of disease

**Figure 3:**
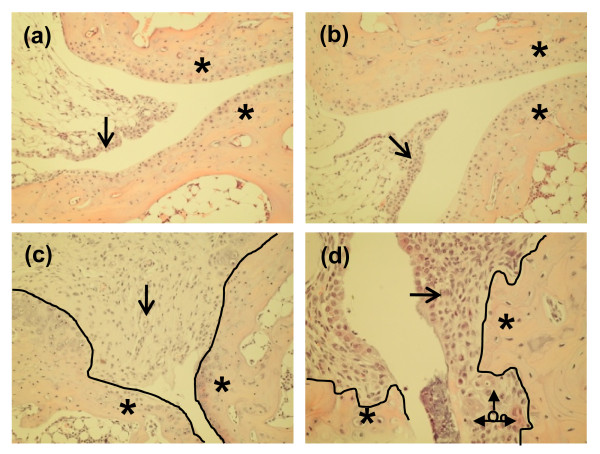
**The disease visual score correlates with histological changes in the affected joints**. **(a) **A representation of ankle joint in wild type mouse at 10 weeks of age showing no evidence of synovitis or bone erosion; **(b) **A representation of a non-inflamed ankle joint in Balb/c IL1ra-/- mouse at 10 weeks of age showing no evidence of synovitis and bone erosion; **(c) **A representation of inflamed ankle joint in Balb/c IL1ra-/- one week after appearance of visual swelling of the joint demonstrating extensive infiltration of the synovium by inflammatory cells and pannus, like synovial proliferation but no bone erosion; **(d) **A representation of inflamed ankle joint in Balb/c IL1ra-/- mouse affected by swelling of the ankle for five weeks as determined by visual inspection, showing synovitis and erosion of bone by osteoclasts (Oc) within the pannus-like synovial proliferation. Black arrows indicate synovium, * joint surface, a continuous black line demonstrates the interface between the synovium and bone and joint surfaces. All figures are of haematoxylin and eosin-stained paraffin-embedded sections, original magnifications ×200.

Having seen changes in mesenchymal progenitors before disease onset and considering that cells with properties of mesenchymal progenitors have been found increased in numbers in the synovium of patients affected by RA, we determined whether mesenchymal progenitors in the BM were affected by the onset of RA-like arthritis in Balb/c IL1ra-/- and with progression of the disease. To achieve this, animals were analysed for the number of mesenchymal progenitors one and five weeks after identification of joint swelling by visual inspection. Histology of the joints was carried out retrospectively to confirm that signs of inflammation resembling those in RA were present and matched the visual score (Figure 3). As expected the histology of ankle joints showed normal synovial membrane in wild type animals (Figure 3a). Joints of Balb/c IL1ra-/- mice, which were scored as not affected by visual inspection also showed a synovial membrane comparable to wild type animals (Figure 3b). In contrast, proliferation of synovial cells with significant inflammatory cellular infiltrations was seen in ankle joints of Balb/c IL1ra-/- mice sacrificed one week from visual scoring of joint disease (Figure 3c). These features became more pronounced with disease progression and were accompanied by pannus invasion, cartilage damage and periarticular bone erosion in mice which had the disease for five weeks as verified from visual identification of joint swelling (Figure 3d).

**Figure 4:**
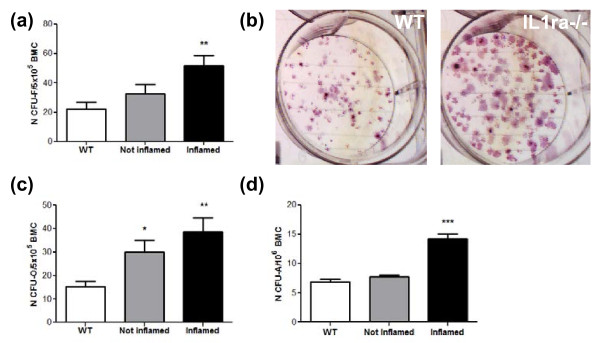
**Increase in bone marrow mesenchymal progenitors at the onset of RA-like disease**. A significant increase in BM-MSC **(a)**, progenitors with osteogenic **(b-c) **and adipogenic **(d) **potential was found in Balb/c IL-1ra-/- mice compared to WT controls at the onset of disease (*n *= 5 with disease in adjacent joint, *n *= 7 with disease in non-adjecent joint). (b) is a representative example of CFU-O cultures from bone marrow cells of Balb/c IL1ra-/- mice affected by RA and age matched WT control. All data are presented as means ± SEM and analysed by one way ANOVA and Bonferroni post-test. *P < 0.05, **P < 0.01, ***P < 0.001. WT, wild type mice; CFU-F, colony forming unit fibroblast; CFU-O, colony forming unit osteoblast; CFU-A, colony forming unit adipocyte; BM, bone marrow.

To determine the number of mesenchymal progenitors at the onset of RA-like arthritis, we measured the number of CFU-F, CFU-A and CFU-O one week after the visual appearance of redness and swelling of the joint. As a number of animals developed asymmetric disease at the knee joints, we compared the number of progenitors present in the bone marrow of femurs, which were adjacent (*n *= 5) or not adjacent (*n *= 7) to an inflamed joint, to age-matched wild type controls (15 to 26 weeks of age, *n *= 7). An increase in CFU-F (Figure 4a), CFU-O (Figure 4b, c) and CFU-A (Figure 4d) was seen in IL1ra-/- mice developing the disease. However, this reached statistical significance only when the mesenchymal progenitors were contained in the bone marrow of a femur adjacent to an inflamed joint. This data suggest that an increase in the number of mesenchymal progenitors occurs at the onset of RA-like arthritis but is more prominent in the bone marrow adjacent to an inflamed joint.

**Figure 5:**
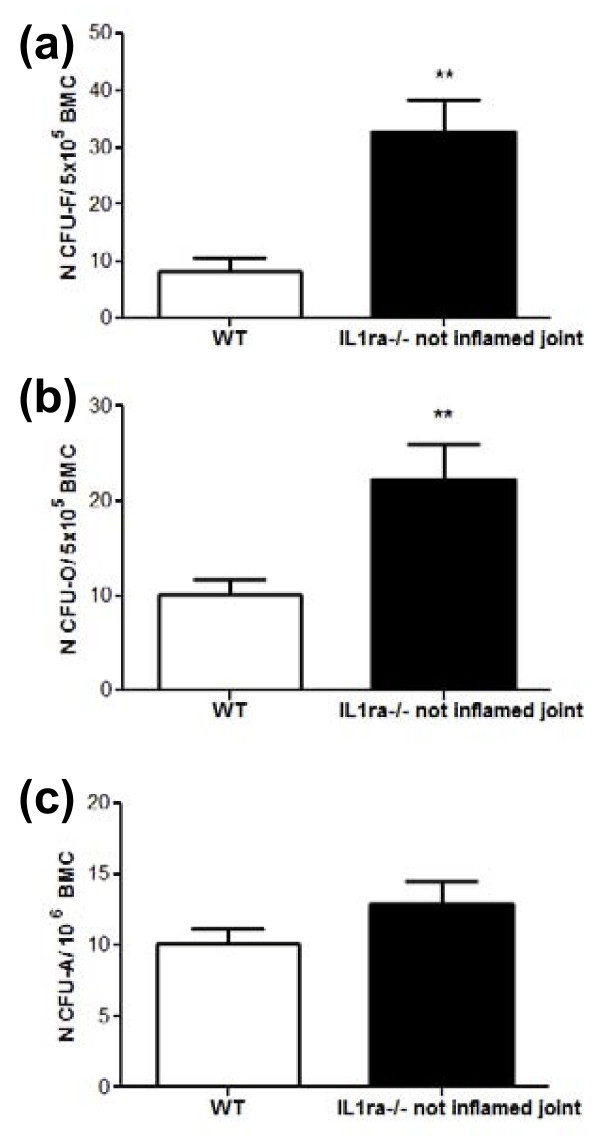
**The increase in mesenchymal progenitors is independent of the site of inflammation with disease progression**. **(a) **A significant increase in the number of CFU-F, independent of the proximity to an inflamed joint, was found in the bone marrow of Balb/c IL-1ra-/- mice compared to WT controls with disease progression (*n *= 8). A significant increase in the number of progenitors with osteogenic **(b) **(*n *= 8) and adipogenic **(c) **(*n *= 8) potential in the bone marrow not adjacent to an inflamed joint in Balb/c IL1 ra-/- mice compared to WT age matched controls. Mice showed signs of RA-like disease for five weeks. All data are presented as means ± SEM and analysed one way ANOVA and Bonferroni post-test. *P < 0.05, **P < 0.01, ***P < 0.001. WT, wild type mice; CFU-F, Colony Forming Unit - Fibroblast; CFU-O, Colony Forming Unit - Osteoblast; CFU-A, Colony Forming Unit - Adipocyte; BM, bone marrow.

To determine whether the number of progenitors significantly increased independently of the site of inflammation with disease progression, femurs not adjacent to the site of inflammation were analysed five weeks after visual appearance of swelling of the ankle joints (age 14 to 23 weeks). A significant increase in the number of CFU-F (Figure 5a, *n *= 8, *P *= 0.001) and CFU-O (Figure 5b, *n *= 8, P = 0.01) was observed independent of the site of inflammation. No significant increase was observed when comparing the number of CFU-A (Figure 5c, *n *= 8, *P *= 0.15). However, considering that Balb/c IL1ra-/- mice showed a significantly reduced number of CFU-A before disease onset, the data indicated an overall increase in the number of CFU-A in the bone marrow of Balb/c IL1ra-/- as a result of the disease. These data suggest that with disease progression the number of mesenchymal progenitors increase independent of the site of inflammation.

### Block in osteoblast differentiation with disease progression

**Figure 6:**
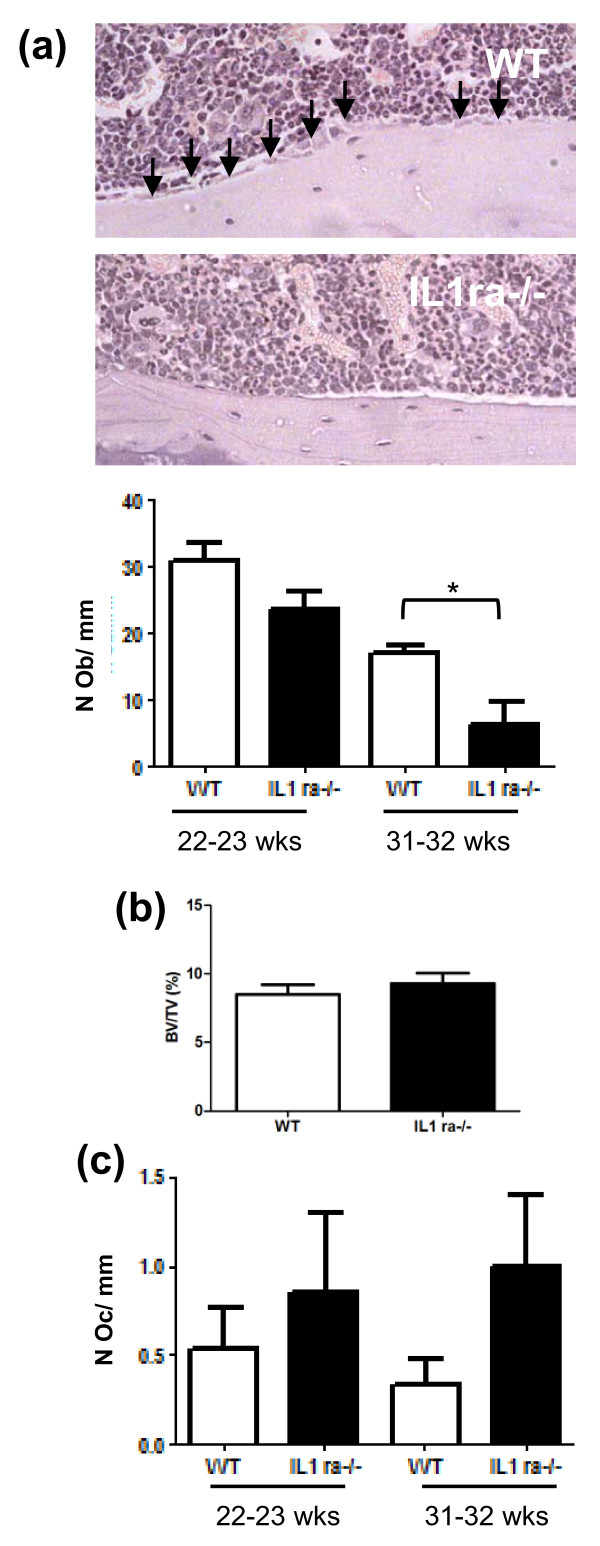
**Decreased osteoblast differentiation is progressive with disease development and age of the mice**. **(a) **A representative example of histological section of osteoblasts at the tibial endo-cortical surface of Balb/c IL1ra-/- (32 to 33 weeks old) compared to WT age-matched control mice. A decrease in the number of osteoblasts over 6.5 mm of tibial endo-cortical surface in Balb/c IL1ra-/- affected by the disease for five weeks was observed and was found more prominent in older mice (aged 31 to 32 weeks, *n *= 3) than younger mice (aged 22 to 23 weeks, *n *= 5) despite they both had the disease for the same length of time. **(b) **X-ray micro CT scanning of tibia of Balb/c IL1ra-/- mice showed no significant difference in bone volume in the cancellous bone area with disease progression (*n *= 8). **(c) **No significant difference in osteoclast numbers was observed with the progression of disease in Balb/c IL1ra-/- mice which were 22 to 23 weeks of age (*n *= 5) or 31 to 32 weeks of the age (*n *= 3) compared to age matched WT control mice. All data are presented as means ± SEM and analysed by unpaired t test. *P < 0.05. WT, wild type mice; BV/TV, bone volume/tissue volume; Oc, osteoclasts; Ob, osteoblasts.

To determine whether an increase in mesenchymal progenitors, particularly progenitors with osteogenic potential, translated into an increase in the number of osteoblasts and bone mass, these were analysed by histomorphometry and microCT analysis. No significant difference was found in the number of osteoblasts at disease onset (23.2 ± 3.2 vs 18 ± 4.1 N Ob/mm, WT vs Balb/c IL1ra-/- respectively, *n *= 6, *P *= 0.71). Interestingly, when mice had the disease for five weeks the number of osteoblasts decreased and this was more pronounced in older mice (Figure 6a, 31 to 32 weeks old, *n *= 3, *P *= 0.039) compared to younger mice (Figure 6a, 22 to 23 weeks old, *n *= 5, *P *= 0.07). To determine whether the decrease in osteoblasts translated into a decrease in bone mass, micro CT analysis was carried out and showed no significant difference when comparing the tibia of Balb/c IL1ra-/- to wild type age-matched mice (15 to 26 weeks of age) at the onset of RA-like arthritis (2.7 ± 0.8 vs 2 ± 0.6, WT vs Balb/c IL1ra-/-, *n *= 8, *P *= 0.14) or with disease progression (Figure 6b, *n *= 8, *P *= 0.50, age 22 to 32 weeks old). Adjacency of the site of inflammation to the analysed tibia did not have any influence on bone mass (data not shown).

As osteoclasts were significantly decreased in Balb/c IL1ra-/- before the start of RA-like arthritis and they are known to be increasingly activated in the joint of patients affected by RA we determined whether the number of activated osteoclasts was affected by the disease onset and progression. No significant difference in osteoclasts numbers was found at disease onset (1.3 ± 0.2 vs 1 ± 0.3 N Oc/mm, WT vs Balb/c IL1ra-/- respectively *n *= 6, *P *= 0.36) or with disease progression in Balb/c IL1ra-/- mice at 22 to 23 weeks of age (Figure 6c, *n *= 5, *P *= 0.54) or 31 to 32 weeks of age (Figure 6c, *n *= 3, *P *= 0.19) compared to age-matched wild type controls. However, as IL1ra-/- mice showed a decreased number of osteoclasts before disease onset, this suggests a change in the activation profile of osteoclasts with disease establishment. Activated osteoclasts are no longer decreased in number compared to wild type mice and, if anything, a trend to increase is seen. Similarly the increase in bone mass seen before disease onset is also lost with disease establishment. This, together with a block in differentiation of the mesenchymal progenitors with osteogenic potential to the osteoblast lineage, suggests an overall accelerated bone loss in Balb/c IL1 ra-/- with disease onset and progression.

## Discussion

The role of the bone marrow niche and mesenchymal stem cell in haemopoiesis and regulation of inflammation is becoming progressively recognised [14-16]. Despite the fact that in RA there are clearly defined disturbances in the haemopoietic and immune system, changes in bone marrow mesenchymal stem cells and their progeny, which may drive those modifications, have not been studied. To address this question we have used the IL1ra-/- on a Balb/c background. This model has histopathological features, which closely resemble arthritis present in patients affected by RA with synovitis, inflammatory cellular infiltration, which includes lymphocytes, pannus formation and periarticular bone erosions. The knocking out of the interleukin-1 receptor antagonist is permissive but not solely responsible for the occurrence of RA-like arthropathy as the occurrence depends also on the mouse strain. Moreover, the histopathological features of the disease differs from those described in patients who harbour a deletion of the interleukin-1 receptor antagonist gene recently described [17]. In those patients there is multiple chronic osteomyelitis, and bone erosions are intraosseous and not periarticular as seen in our murine model or in patients affected by RA. Therefore, the Balb/c IL1ra-/- is a valuable model to study events which occur over time and in the BM. Obtaining BM samples from patients affected by RA and who have not undergone treatment is difficult.

Here, for the first time, we provide evidence of changes in the composition of the bone marrow niche present already before the development of RA-like disease. We have shown a deficiency in mesenchymal progenitors with adipogenic potential and bone marrow adiposity together with a decrease in the maturation of osteoclasts before disease development. Moreover, this was present only in Balb/c IL1 ra-/- mice, who progressed to develop RA-like disease and not in C57BL6 IL1ra-/-, which have been shown not to develop RA-like arthritis, suggesting an association of these abnormalities with RA development. Although it is difficult to predict the precise cascade of events leading from low adipogenesis to decreased activation of osteoclasts, there is evidence that the two processes may be linked. Mature adipocytes release adipokines such as leptin, ghrelin and adiponectin, or growth factors such as monocytes chemotactic protein 1 (MCP-1) [18]. For examples, MCP-1 has been shown to mediate osteoclastogenesis and bone resorption [19]. Further studies are now needed to understand the genetic alterations and the cascade of events leading to those abnormalities.

Regardless of the precise cascade of events, our data are in agreement with other published data. In the study by Naivares *et al *(2009) low bone marrow adiposity has been positively correlated with the ability to support proliferation of hematopoietic progenitors [8]. Lack of adipogenesis in AZIP/F1 recipient mice (genetically incapable of forming adipocytes) enhanced hematopoietic recovery after lethal irradiation by enhancing engraftment of short term progenitors [8]. Adipocyte-rich marrow was shown to harbour a decreased frequency of progenitors and relatively quiescent stem cells [8]. Therefore the reduction in adipogenesis in Balb/c IL1ra-/- mice supports the notion of an enhanced stimulation of the HSC to proliferate and is in agreement with the study by Schonland *et al. *(2003) where individuals with the HLA-DRB104 haplotype, shown to be more at risk for developing rheumatoid arthritis, had accelerated telomere shortening of both lymphocytes and neutrophils, a marker of cellular proliferation and ageing of haemopoietic progenitors [4].

Dysregulated adipogenesis may be responsible for the establishment of a proinflammatory environment. Adiponectin has been suggested to have a role *in vivo *for immediate moderation of inflammatory reactions occurring after invasion by foreign pathogens and prevents an excessive and prolonged inflammatory response. Indeed lipopolysaccharide (LPS) induced expression of TNFα mRNA in macrophages and was markedly suppressed by pre-treatment with recombinant adiponectin [20]. Adiponectin pre-treated macrophages failed to release TNF-α in response to LPS [20]. Therefore it is not unreasonable to suggest that the low number of adipocytes found in the bone marrow of Balb/c IL1ra-/- mice may lead to reduced levels of adipokines, such as adiponectin, which in turn reduce activation of macrophages and allows increased presence of inflammatory cytokines such as TNF-α, following the start of any inflammatory process. Indeed, elevated levels of TNFRII, a surrogate soluble receptor, which parallel levels of TNF-α, has been found elevated in the preclinical phase of disease of patients that go on to develop RA up to eight years after [21].

MSC share many properties with fibroblast-like synoviocytes (FLS) [22], the cells responsible for the thickening of the synovium in the joint in RA and perpetuation of the inflammatory process by recruiting and supporting the survival of inflammatory cells [23]. It is thought that MSC may even contribute to the FLS pool in the joint. When the bone marrow of green fluorescence protein (GFP) transgenic donor mice was transplanted into lethally irradiated recipient mice, it was shown that normal FLS contained a minor fraction (1.2%) of GFP+ cells [24]. As FLS undergo increased proliferation it is not unreasonable to suggest that MSC may undergo expansion in the bone marrow too at disease onset and change the composition of the bone marrow niche. Our data point to increased numbers of MSC following establishment of the disease. Our findings are not in agreement with a previous study measuring the number of bone marrow mesenchymal progenitors in patients affected by RA, where no significant difference was found [25]. The reason for the apparent discrepancy may lie in the fact that most patients included in the study by Kastrinaki *et al*. (2008) [25] had undergone treatment and those who had not undergone treatment were a very small number with a wide age range. This may have compromised the detection of any difference, especially considering that the number of CFU-F tend to decrease with age [12].

Of interest is the accumulation of progenitors, which are unable to differentiate to the osteoblast lineage. The mechanism leading to MSC accumulation and blocking of their differentiation are unclear and likely due to a complex interplay of cytokines and cellular cross talks, mostly related to the *in vivo *inflammatory environment. When these cells were induced to differentiate to the osteoblast lineage in vitro they showed formation of colony forming unit-osteoblasts, which were alkaline phosphatase positive and did not differ in the pattern of staining from those derived from the bone marrow of their wild type age-matched controls. Proinflammatory cytokines such as TNF-α and IL1β have been shown to block osteogenic differentiation *in vitro *[26,27] and support our findings. The accumulation of MSC, known to secrete RANKL, may also lend an explanation to the loss of defective osteoclastogenesis observed in the Balb/c IL1ra-/- animals before disease onset. Indeed, our data shows that with the establishment of disease the number of osteoclasts is no longer significantly decreased and, if anything, it exhibits a trend to increase. This is in agreement with the well-known increase in the number of activated osteoclasts reported in the joints of patients with RA and with data suggesting that at very early stages of inflammation, when levels of TNF-α are low, mesenchymal stem cells are required for osteoclastogenesis [28]. All together our data are in agreement with reports of RA patients developing systemic osteoporosis with time [29] and of systemic accelerated bone loss in another model of inflammatory arthritis mainly as the result of arrested osteoblast function [30]. Ways to induce effective differentiation of the mesenchymal progenitors to the osteoblast lineage may provide both a way to reduce expansion of MSC and prevent systemic bone loss.

The accumulation of MSC raises the intriguing question whether these cells, known for their immunosuppressive properties, are exerting the expected anti-inflammatory action and whether the proposed administration of additional MSC, as novel therapeutic strategy in RA, would have the desired effect of dampening the auto-immune responses. Despite a wealth of literature describing the immunosuppressive ability of MSC, more recently a pro-inflammatory phenotype of MSC has also been described [31,32]. This requires activation of TLR4 and possibly TLR3 receptors present on MSC. Indeed overexpression of TLR3 and TLR4 has been described in synovial fibroblasts of patients affected by RA and beg the question whether bone marrow mesenchymal stem cells in RA patients show similar overexpression and have a pro-inflammatory phenotype as a result of their engagement [33]. The fact that administration of murine MSC in models of RA disease has given contrasting results [34,35] highlights that this is a potential problem and calls for a closer analysis of the inflammatory phenotype of the patients' MSC at the time of active disease, following expansion before administration and after administration to the patient. This may help to determine whether MSC preparation varies in terms of predisposition to acquire a pro-inflammatory phenotype or whether this is an acquired characteristic as a result of contact with the patients' cellular environment. Moreover, it may establish whether the cellular environment confers the pro-inflammatory phenotype depending on the disease stage and status of remission.

## Conclusions

We have shown an accumulation of MSC progenitors with the onset of RA-like disease and a block in their differentiation to osteoblasts. These data highlight the need for therapeutic intervention with molecules capable of inducing differentiation of MSC to osteoblasts to prevent the onset of osteoporosis. Moreover, it puts into question whether additional administration of MSC proposed as treatment to reduce the autoimmune response [30] is required and would be efficacious. More studies into the molecular mechanism leading to enhanced maintenance of MSC in RA-like disease, their pro-inflammatory phenotype and ways to interferes with this are required. Small molecules such as glycogen synthase kinase -3 inhibitors have been shown to have anti-inflammatory properties and to drive commitment of MSC to osteogenic lineage [36,37], suggesting they may have a therapeutic impact in both reducing inflammation and bone loss.

## Abbreviations

ALP: alkaline phosphatase; Balb/c IL1ra-/-: Interleukin-1 receptor antagonist knock out mouse on a Balb/c background; BM: bone marrow; BMC: bone marrow cells; CFU-A: Colony Forming Unit-Adipocyte; CFU-F: Colony Forming Unit-Fibroblasts; CFU-O: Colony Forming Unit-Osteoblast; DMEM: Dulbecco's modified Eagle medium; FCS: fetal calf serum; FLS: fibroblast-like synoviocytes; GFP: green fluorescence protein; HSC: haemopoietic stem cell; LPS: lipopolysaccharide; MCP-1: monocytes chemotactic protein 1; MSC: mesenchymal stem cells; RA: rheumatoid arthritis; TRAP: tartrate resistant acid phosphatise; WT: wild type mice.

## Competing interests

The authors declare that they have no competing interests.

## Authors' contributions

STM, LK, AG, LC and DH contributed to the collection and/or assembly of data, data analysis, and to critical revision and final approval of the manuscript. MJN contributed to conception and design, critical revision and final approval of the manuscript. AGW and PIC contributed to interpretation of data, writing of the manuscript, and final approval of the manuscript. IB contributed to the conception and design of the study, data analysis and interpretation, manuscript writing, and revision and final approval of the manuscript.
